# Assessment of Network Inference Methods: How to Cope with an Underdetermined Problem

**DOI:** 10.1371/journal.pone.0090481

**Published:** 2014-03-06

**Authors:** Caroline Siegenthaler, Rudiyanto Gunawan

**Affiliations:** Institute for Chemical and Bioengineering, Swiss Federal Institute of Technology Zurich, Zurich, Switzerland; National Institute of Genomic Medicine, Mexico

## Abstract

The inference of biological networks is an active research area in the field of systems biology. The number of network inference algorithms has grown tremendously in the last decade, underlining the importance of a fair assessment and comparison among these methods. Current assessments of the performance of an inference method typically involve the application of the algorithm to benchmark datasets and the comparison of the network predictions against the gold standard or reference networks. While the network inference problem is often deemed underdetermined, implying that the inference problem does not have a (unique) solution, the consequences of such an attribute have not been rigorously taken into consideration. Here, we propose a new procedure for assessing the performance of gene regulatory network (GRN) inference methods. The procedure takes into account the underdetermined nature of the inference problem, in which gene regulatory interactions that are inferable or non-inferable are determined based on causal inference. The assessment relies on a new definition of the confusion matrix, which excludes errors associated with non-inferable gene regulations. For demonstration purposes, the proposed assessment procedure is applied to the DREAM 4 In Silico Network Challenge. The results show a marked change in the ranking of participating methods when taking network inferability into account.

## Introduction

The construction of biological interaction networks with the goal of uncovering causal relationships between genotype and phenotype constitutes a major research topic in systems biology [1]. The inference of GRNs from gene expression data is part of research activities in this area. At the time of writing, a search of the keywords “gene” and “network inference” on the pubmed literature database (excluding review articles) returned more than 200 results since the year 2000. Because of the rapid increase in the number of methods created for GRN inference, each with distinct advantages and disadvantages, a fair assessment of the effectiveness of the methods in practice is indispensable.

A rigorous methodology for an objective assessment of biological network reconstruction algorithms has been presented within the Dialogue on Reverse Engineering Assessment and Methods (DREAM) project [2]–[6]. Community-wide challenges on biological network inference are organized on an annual basis, where the inference of GRNs has been a recurring topic. In the past GRN inference challenges, participants applied a network inference method of their choice to datasets provided by the organizers, and submitted an ordered list of gene regulatory interactions according to the likelihood of their existence. The assessment of network predictions involved a comparison with the reference or gold standard network, by evaluating the confusion matrix (i.e. the numbers of true positives/negatives and false positives/negatives) [3], [5], [6]. The submissions were then scored and ranked using the area under the curves of the receiver operating characteristics (ROC) or precision-recall, denoted by AUROC and AUPR, respectively [3], [5]–[10]. It is important to note that this assessment procedure relied on the implicit assumption that the GRN could be uniquely inferred from the data.

A network inference problem is underdetermined when the experimental data do not contain the necessary information for reconstructing the complete network. In such a case, the network is deemed non-inferable. The inference of GRNs from the typical gene expression data (e.g. from knock-out, knock-down experiments) has often been stated to be underdetermined [6], [11], [12], but this claim has not yet been investigated rigorously. There exist many factors that could lead to an underdetermined inference problem, such as errors during the experiments, noise in the data, and the number and types of network gene perturbation experiments. As a consequence, some gene interactions cannot be inferred and more than one network can agree with the data.

In the present work, we emphasize the importance of network inferability in assessing the performance of GRN inference methods. We present an assessment procedure that incorporates the inferability of gene regulatory interactions by redefining the confusion matrix. The novelty in the new assessment is that inference methods are not penalized for mis-identifying non-inferable interactions. As a demonstration, we apply the new performance assessment to the submissions of the DREAM 4 In Silico Network Challenge and compare the outcome with the original performance scores.

## Materials and Methods

### DREAM 4 In Silico Network Challenge

#### Challenge description

The DREAM project is an international initiative with the aim to better understand the strengths and weaknesses of methodologies for reverse-engineering of biological networks and to foster improvements in this research area. Challenges in the area of biomolecular network inference and quantitative modeling in systems biology are organized on a yearly basis, in which participants are provided with experimental data and invited to submit predictions of network structures (or parameters) using an inference algorithm of their choice. These submissions are subsequently scored and ranked based on how well the predictions agree with the gold standard or reference network.

In the DREAM 4 In Silico Network Challenge [13], the main task consisted of inferring GRN structures from simulated steady-state and time-series gene expression data. In the first and second subchallenges, data of steady-state mRNA levels were provided for wild-type and for gene knock-out (KO) and gene knock-down (KD) experiments, for five 10-gene and five 100-gene GRNs. For wild-type, time-series mRNA expressions were also provided. In addition, for the 10-gene subchallenge, expression data from multifactorial gene perturbation experiments were made available. Participants submitted a ranked list of gene interactions, ordered according to the confidence in their existence. A total of 29 predictions were submitted to the first (10-gene) subchallenge and 19 predictions to the second (100-gene) subchallenge.

#### Gold standard networks and datasets

The gold standard networks were constructed by extracting subnetworks from transcriptional regulatory networks of *Escherichia coli* and *Saccharomyces cerevisiae* using GeneNetWeaver (GNW, version 2.0) [14]. While cycles represented a motif of interest, auto-regulatory interactions were omitted from the gold standard networks. A detailed kinetic model in the form of stochastic differential equations (SDEs) was created for the generation of synthetic gene expression data. Measurement noise was added to the simulated data using a noise model of microarray data, resembling a mixture of normal and lognormal noise distribution [14]. While the kinetic model described the dynamics of mRNA and protein concentrations, the participants received only mRNA expression levels.

Simulations of the SDE model provided the expression data of the wild-type network (unperturbed network). Single-gene KO experiments involved setting the mRNA transcription rate of a particular gene to zero. Similarly, single-gene KD data were simulated by halving the transcription rate of a gene. Participants were also provided with multifactorial perturbation (10-gene subchallenge only) and time-series data, in which the basal activations of all or a third of the genes, respectively, were perturbed by random amounts. For more details concerning the datasets in this challenge, we refer to [13].

#### DREAM assessment procedure

The scoring of submissions in the DREAM network inference challenges has been described in detail elsewhere [3], [5], [6]. Briefly, for each submission, a series of network structures of increasing size is generated by sequentially adding entries from the list of gene interactions, one at a time. The 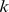-th network of this series thus includes the first 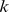 gene interactions in the submitted list. Subsequently, for each network structure, a confusion matrix is computed with respect to the gold standard network, providing the numbers of true positives (TP(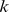)), true negatives (TN(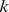)), false positives (FP(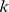)) and false negatives (FN(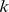)). A TP describes a correct prediction of an edge in the gold standard network, while a FP occurs when a predicted edge does not belong to the gold standard network. Furthermore, a TN refers to an edge that neither belongs to the prediction nor to the gold standard network, while a FN corresponds to any edge in the gold standard that is missing in the predicted network. The scoring of teams is based on the confusion matrices and the performance metrics in Equation (1). P corresponds to the number of gene regulatory interactions in the gold standard network, while N is the number of gene interactions that are possible, but not in the gold standard network. Participants are finally ranked according to how well their predictions agree with the gold standard networks when compared to random network predictions, using the AUROC and AUPR [3], [5].
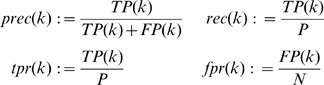
(1)


### New assessment procedure incorporating GRN inferability

#### Basics of graph theory

In the following, we introduce the necessary graph theoretical nomenclatures, which will be used to develop the new assessment procedure. A *graph* is an ordered pair 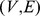, where 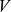 is the set of 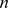 nodes and 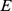 is the set of 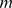 edges. An edge 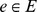 is defined by the pair of nodes 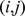, which in turn are incident to the edge 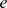. A *directed edge* is defined by the ordered pair 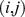, describing an edge from node 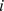 pointing to node 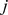. A *directed graph* (digraph) is a graph in which all of its edges are directed. Accordingly, a *directed path* consists of a sequence of nodes such that there exists a directed edge from one node to the next. If there exists a directed path from node 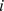 to node 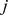, then 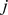 is said to be accessible from 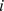. A *directed cycle* is a directed path in which the starting and ending nodes are the same. Finally, a *directed acyclic graph* (DAG) is a digraph without any directed cycle.

A strongly connected component or *strong component* of a digraph 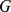 is a maximal subset of nodes in 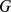, where any two nodes in the subset are mutually accessible. All nodes of a directed cycle belong to the same strong component, while each node that is not part of any cycle, is a strong component of its own. The *condensation* of a digraph 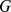 is the DAG of the strong components of 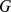, which can be constructed by lumping nodes together that belong to the same cycle [15].

The *transitive closure* of a digraph 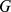, denoted by 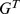, is the digraph which possesses the edge 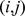, whenever node 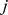 is accessible from node 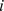. In general, more than one digraph can share the same transitive closure, and the set of such digraphs is denoted by 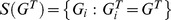. Meanwhile, the *transitive reduction* of 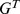, denoted by 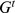, is defined as the most parsimonious member of 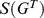 (i.e. the graph with the fewest edges). The transitive reduction of a DAG is unique and given by 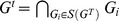 [16]. For DAGs, the transitive reduction can be obtained by recursively pruning any directed edge 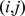, whenever a directed path from 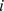 to 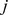 exists that does not include 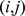 [15]. In contrast to DAGs, the transitive reduction of a digraph may not be unique.

#### Inferability of GRNs

A GRN describes the causal relationships among genes, which are often represented using a digraph. A directed edge from gene 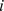 to gene 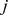 (denoted 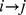) implies that the expression of gene 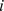 influences the expression of gene 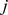. The inference of GRNs thus involves more than finding associations among genes. Such a problem is inherently related to causal inference [17]. To date, the inference of causal connections in a biological network remains an unsolved problem due to its underdetermined nature, implying that the network structure cannot be uniquely inferred.

In the following, a GRN is deemed inferable, when a unique digraph exists that agrees with the provided experimental data. An underdetermined inference problem immediately implies that the network is not completely inferable. Note that network inferability depends on the available information contained in the data, but not on the inference method. Data informativeness is affected by several factors, including experimental conditions, experimental errors, and data noise. The study of causal inference has shown that causality can only be inferred from observations of perturbation experiments (interventions) in which the perturbations are known [17]. In the DREAM 4 In Silico Network Challenge, single-gene KO and KD are examples of such intervention experiments, and thus the data possess relevant information for establishing causal relationships among genes. Meanwhile, the causal information in multifactorial and time-series data is difficult to determine, and the extraction of this information depends on additional background knowledge and assumptions. In this study, we consider only the KO/KD experiments in determining the inferability of causal edges.

The data from multifactorial perturbations in the DREAM 4 challenge can be classified as observational data. Inferring the true causal graph with observational data is typically underdetermined, even when simplifying assumptions, such as the causal Markov assumption and the causal faithfulness assumption, are satisfied and the observed variables are causally sufficient [18]. Correspondingly, there exists not one, but a set of graphs that are consistent with the data and the assumptions. A possible approach to determine such a set of graphs may be based on the concept of Markov equivalence class [19]. Nevertheless, in certain scenarios where sufficient background domain knowledge exists and the number of variables is small, causal inference from observational data may be possible.

On the other hand, time-series data are particularly relevant for inference methods where the GRN is modeled as a dynamical system. The complete description of such a model requires not only the structure (topology) of the GRN, but also the kinetic functions of the gene-gene interactions. The identification of network structure from time-series data is usually formulated as an optimization problem to minimize model prediction errors. However, even when the data are ideal (noise-free and complete), the experimental conditions are given, and the kinetic equations are known, the optimization above is challenging and often produce multiple, indistinguishable solutions[20], [21]. The distinguishability issue is equivalent to network (non-)inferability described above, which can be alleviated by enforcing additional assumptions or constraints regarding the GRN [21].

One domain that is strongly affected by network (non-)inferability is the performance assessment of inference methods. When a network or part of a network is not inferable, no algorithm will be able to accurately infer the full network structure. Therefore, a fair assessment should not penalize any incorrect prediction of non-inferable parts of the network. While the underdetermined nature has been widely recognized as a stumbling block in the inference of GRNs, the issue related to network inferability has not been explicitly taken into consideration in existing assessments.

#### Assessment of GRN inference methods: Coping with an underdetermined problem

**Figure 1:**
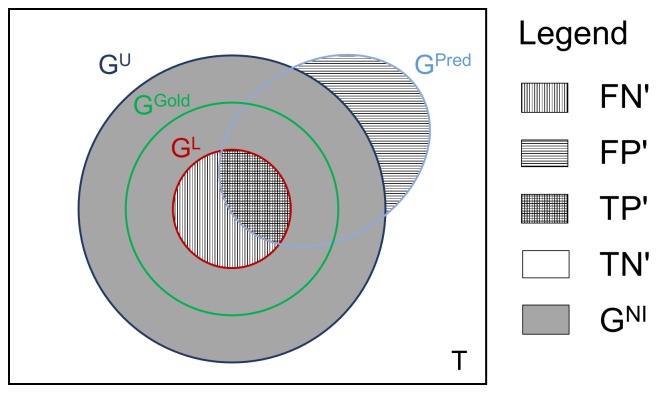
Venn diagram of confusion matrix incorporating network inferability. 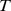
 denotes the set of all possible (directed) edges among nodes in the network. FN  =  false negative. FP  =  false positive. TP  =  true positive. TN  =  true negative. 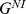  =  non-inferable edges.

In order to cope with the underdetermined nature of GRN inference in assessing the performance of network inference methods, we propose a new definition of the confusion matrix as depicted in the Venn diagram in Figure 1 and described in Equation (2). The set of all possible edges is denoted by 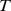 and the set of edges in the gold standard network is denoted by 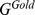. Given a dataset of differential gene expression profiles, the set 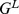 contains edges in 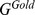 whose existence can in principle be confirmed. Meanwhile, the set 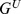 is defined such that 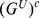 (i.e. the complement of 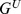) consists of edges that can be validated by the data to be an element of 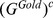. Here, the non-inferable edges 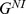 are defined as edges whose existence can neither be confirmed nor refuted by the data. Thus, we have the simple relationship 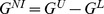. The set 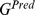 denotes the set of edges in the predicted network structure. The basic idea of the new assessment is to exclude edges that belong to 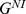 from the calculation of the confusion matrix. Below, we describe a procedure for determining the sets 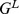 and 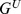 for the DREAM 4 network inference challenge. The algorithm for constructing 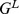 and 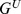 in a more general setting is currently in progress (SMM Ud-Dean and R. Gunawan, unpublished).
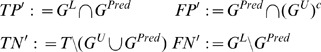
(2)


Following the arguments from causal inference described above, the inferability of GRNs in the two DREAM 4 subchallenges is determined based on single-gene KO/KD experiments. In an ideal scenario, knocking-out or knocking-down a gene should lead to (steady-state) differential expression among all genes that are either directly or indirectly regulated by the perturbed gene. Unfortunately, it is not possible to discriminate direct and indirect regulations from steady-state data [15]. Since the datasets in the network inference subchallenges include all single-gene KO/KD experiments, the transitive closures of the gold standard networks (i.e. 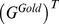) can be constructed in the ideal case, but not the true network structure. In reality, even the transitive closures will have errors due to factors such as data noise and gene compensatory effects.

The transitive closure 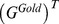 represents the largest network structure, as measured by the number of edges, that is consistent with the gene expression data considered above. In fact, any digraph belonging to the set 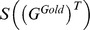 is also in agreement with the data. Thus, the set 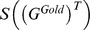 describes the ensemble of networks that cannot be discriminated using steady-state single-gene KO/KD data. Here, we set the upper bound of the ensemble 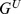 as the smallest digraph that contains 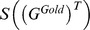, and the lower bound of the ensemble 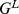 as the largest digraph whose edges are present in every member of 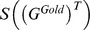. Consequently, the difference between 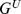 and 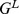 are edges that can neither be rejected nor confirmed from the data. Note that when 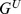 and 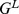 are the same, the GRN is inferable.

From the definition of 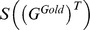, 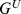 can be set to 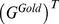. If the GRN can be represented as a DAG, then 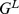 is given by the transitive reduction of 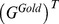. However, the gold standard networks in these subchallenges contain directed cycles. Therefore, 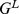 is constructed based on a modified version of the pruning algorithm for transitive reduction described in [15]. Specifically, 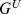 is first condensed to produce a DAG of the strong components. Then, the pruning algorithm is applied to the condensed graph to remove any edge 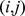 for which a directed path exists from 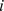 to 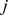 that does not involve 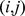. In addition, we also prune edges incident to any strong component associated with a directed cycle. Finally, the strong elements are expanded and edges incident to nodes involved in cycles of more than two nodes are further removed, because the causal relationships among these nodes cannot be inferred from single-gene KO/KD data. The network structure produced by this procedure is set as 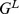.

**Figure 2:**
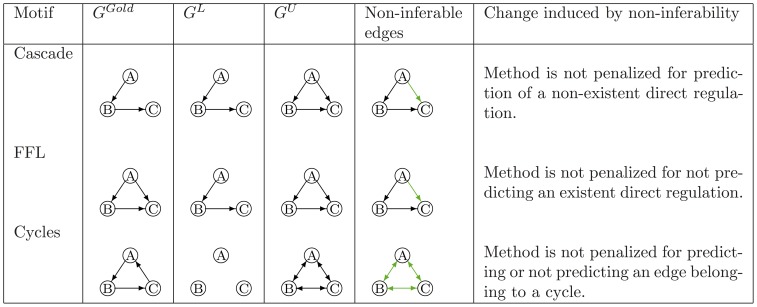
Inferability of network motifs from single-gene KO/KD experiments. The new assessment procedure omits edges that are not inferable from single-gene KO/KD experiments, from the scoring of submissions. Compared to the assessment in the DREAM 4 challenge, FPs and FNs associated with cycles, cascades and FFLs are considered in the new assessment.

In Figure 2, we summarize the differences between the assessment based on the redefined confusion matrix and the original assessment of the DREAM 4 challenge. In the new assessment, FPs and FNs associated with direct/indirect regulations, often referred to as cascade and FFL (feed-forward loop) errors, are omitted in the scoring of submissions. Indeed, cascade and FFL motifs have been identified as one of the important systematic prediction errors in the DREAM network inference challenges [6], [22]. In addition, edges incident to any strong element and those among nodes in directed cycles with more than two nodes are non-inferable from single-gene KO/KD experiments, and thus are not considered in the new assessment.

#### Scoring of network submissions

For each network submission, the AUROC and AUPR were computed based on the redefined confusion matrix in Equation (2). Accordingly, the formulas of precision, recall, true positive rate and false positive rate were adapted (see Equation (3)).
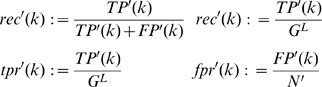
(3)


Following the scoring procedure in DREAM 4, a set of 100′000 random predictions were generated for each gold standard network in the subchallenge. For each random prediction, the AUROC and AUPR were computed using the performance metrics in Equation (3). Subsequently, the area under the curve (AUC) corresponding to a prescribed acceptable p-value (e.g. 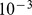) was determined from the random predictions. In this work, the value for 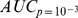 was taken as the 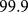-th percentile of the AUC in each set of random networks. Finally, the score for each submitted network was computed based on Equation (4), where the AUC was either AUROC or AUPR. Correspondingly, any submission with an AUROC or AUPR below the acceptable p-value would have a score less than 1 and could be considered as not performing better than random. Using the scoring presented in Equation (4), we avoided fitting a probability density function over the AUROCs and AUPRs of random predictions and estimating (extremely small) p-values of the submissions from such a density function. The final ranking of the participating groups was based on the overall score (see Equation (6)) that incorporated the mean scores for AUROC and AUPR (see Equation (5)), where 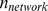 was the number of networks in the evaluation (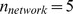 in 10-gene and 100-gene subchallenges). The scoring procedure was implemented in MATLAB (File S1) and more detail of the implementation is provided in the supporting information (Figure S1).
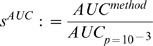
(4)

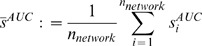
(5)

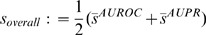
(6)


## Results and Discussion

**Figure 3:**
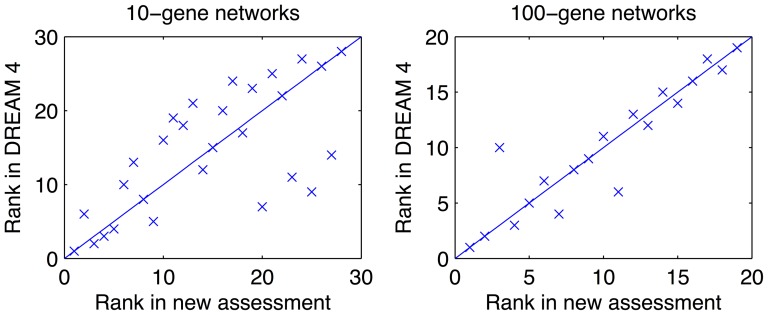
Comparison of the team rankings between the new and the original assessment. The two assessment procedures are applied to the 10-gene subchallenge (left) and the 100-gene subchallenge (right) of the DREAM 4 In Silico Network Challenge.

We applied the new assessment procedure to the network predictions submitted to the 10-gene and the 100-gene subchallenges of the DREAM 4 In Silico Network Challenge (D. Marbach and G. Stolovitzky, private communication, June 2012). One submission to the 10-gene subchallenge was omitted in the comparison below, as the team only provided predictions for 4 out of the 5 networks. The sets 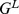 and 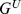 were constructed from the gold standard networks as described above. Table 1 gives the sizes (numbers of edges) of the gold standard networks, the lower and upper bound of the network ensembles, and the number of non-inferable edges. Figure 3 shows the comparison between the team rankings using the new and the original DREAM 4 assessment for the 10-gene and 100-gene subchallenges (for comparison of the performance scores, see Figure S2 and Tables S1-S2). The performance scores in the DREAM 4 assessment were re-computed using a previously published procedure[2].

**Table 1:** Network Sizes in the DREAM 4 In Silico Network Challenge.

Size	Network	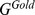	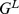	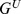	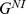
10	1	15	5	21	16
	2	16	2	47	45
	3	15	1	59	58
	4	13	3	40	37
	5	12	5	33	28
100	1	176	93	639	546
	2	249	60	667	607
	3	195	35	2375	2340
	4	211	38	2316	2278
	5	193	60	953	893

The Spearman's rank correlations between the rankings of the DREAM assessment and the new assessment in both subchallenges, given in Table 2, show a significant linear relationship. Indeed, methods that ranked in the top half in the DREAM assessment mostly stayed among the top half in the new assessment. In particular, the best and the worst teams were the same in the two assessments. Nevertheless, the rankings of many teams changed prominently when using the new assessment. For example, in the 10-gene network subchallenge, the second best team in the new ranking was previously ranked 6-th, and the 7-th and 9-th best teams in the original ranking were now among the worst performers (20-th and 25-th, respectively). The two assessments were more consistent with each other in the 100-gene than in the 10-gene network subchallenge, since the 100-gene networks were more sparse than the 10-gene networks. Consequently, the lower bound 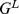 of the 100-gene networks approximated the gold standard networks much better than that of the 10-gene networks. According to Figure 1, the numbers of TP and FN would therefore differ relatively little between the original and new assessments among the 100-gene networks. Similar observations could also be made by comparing the overall team scores (see Figure S2).

**Table 2:** Spearman's rank correlation, two-sided.

Challenge	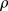	p-value
DREAM 4 size 10	0.69	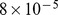
DREAM 4 size 100	0.92	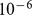

**Figure 4:**
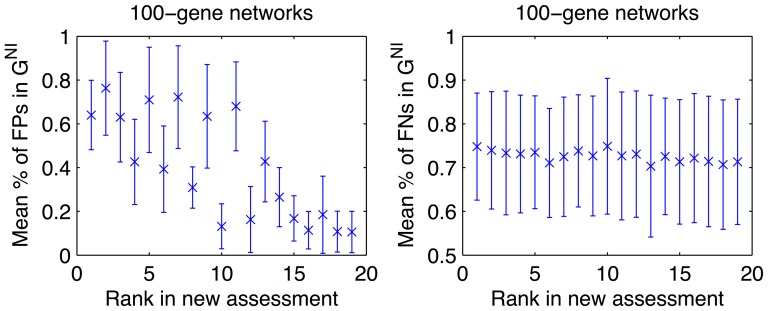
Fractions of FPs (left) and FNs (right) that are non-inferable from single-gene KO/KD experiments. Error bars show standard deviation over five 100-gene networks.

In order to ascertain the extent to which the errors of each inference method could be explained by interactions that were non-inferable, we determined the fractions of FP and FN edges that were non-inferable. Specifically, we created a GRN prediction 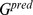 by including edges from each submission list up to the size of the respective gold standard network, and compared the network prediction with the gold standard. The mean fractions of non-inferable FPs and FNs and the standard deviation over the five 100-gene networks are shown in Figure 4 (for individual team fractions, see Tables S3–S4). The mean fractions of non-inferable FNs were quite consistent for all inference methods, lying between 70% and 80%, which was in line with the mean fraction of non-inferable edges within the reference network (71.2%). Interestingly, the mean fraction of non-inferable FPs could segregate good and bad performing methods. As shown in Figure 4, a high fraction of non-inferable FPs (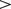
60%) was representative of better performing methods, while a low fraction (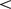
20%) of non-inferable FPs was commonly observed among poorer inference algorithms. Hence, errors (FPs and FNs) of good performing methods could be explained to a great extent by the non-inferable part of the GRN.

In several DREAM network inference challenges, a consensus network constructed from submissions to the challenge has been shown to reliably outperform any individual method [5], [6], [22]. This community strategy takes advantage of the wisdom of crowds by incorporating complementary information extracted by different inference methods, thereby mitigating weaknesses of any particular method [22]. However, as network inferability is independent of the method used in the inference, the consensus network will still be affected by errors associated with non-inferable edges. For illustration purposes, we again used network structures 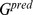 from the submissions to the 100-gene subchallenge as before, and identified FNs that were common among all these network predictions. Table 3 reports the number of common FNs for each reference network in the subchallenge and the percentage of these FNs that were deemed non-inferable (i.e. belonging to 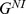). The results indicated that a high fraction of these common errors were associated with non-inferable edges in the gold standard networks. These common errors would naturally appear in the consensus network. Thus, no method, the community strategy included, is expected to be able to predict these edges accurately.

**Table 3:** Common FNs among 100-gene network predictions.

	Net 1	Net 2	Net 3	Net 4	Net 5
Number of common 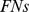	32	78	50	61	65
Fraction of 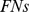 that are non-inferable	56.3%	83.3%	86.0%	75.4%	58.5%

Certain network motifs appeared to be more difficult to infer than others [6], [22]. The inference of some of these motifs, particularly cascade and FFL, involved discriminating direct and indirect regulations. As explained earlier, considering the gene perturbation experiments in the DREAM network inference challenges, the participants did not have the necessary information for such discrimination. Therefore, the systematic errors associated with cascade and FFL motifs should be expected. A fair and relevant assessment of network inference methods would therefore need to incorporate not only network inferability, but also a design of experiments for generating the appropriate datasets. In case of an ideal design of experiments, the performance of inference methods could be assessed without considering network inferability. In practice, network inference methods should carefully consider the limitations in inferring causal interactions from experiments, and data should be treated differently according to the kind of experiment performed.

In the construction of 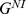, several factors that affect the inferability of gene regulation have not been accounted for. For example, data noise could render the identification of edges difficult, which in turn would increase the number of non-inferable edges. Unfortunately, the particular edges that would become non-inferable due to noise, could not be identified *a priori*. Still, the increase in FP and FN rates could be estimated if the probability distribution of the noise was known. As a good inference method should handle data noise adequately, the exclusion of the impact of noise in determining 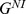 seemed reasonable.

In addition to noise, multifactorial perturbation and time-series experiments were not taken into consideration in constructing 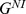. As discussed earlier, data from such experiments could only reveal associations among genes, not causal relationships. Nevertheless, gene associations could still be used for confirming or rejecting edges, even though the direction of the edges cannot be determined. Furthermore, the nature of the gene regulations, i.e. whether a gene activates or inhibits the expression of another gene, has been ignored. Certain regulatory motifs, such as coherent and incoherent FFLs, might therefore be more difficult to infer from from single gene KO/KD experiments [23]. Also, gene compensatory effects, where the effects of perturbing a gene could be masked by other regulator genes, would add further complications, namely in the inference of the fan-in motifs. While all of these factors would certainly enlarge the non-inferability part of the GRN, the 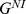 described above should provide a reasonable, albeit pessimistic, approximation of the non-inferable edges that need to be excluded from the assessment.

## Conclusion

In this study, we presented a new performance assessment for GRN inference methods. The rationale of the proposed assessment was that the performance of an inference algorithm should only be evaluated based on parts of the network that were inferable from the experiments performed. The inferability of GRNs was analyzed based on the causal information that could be extracted from gene perturbation experiments. The DREAM 4 In Silico Network Inference Challenge, particularly the 10-gene and 100-gene subchallenges, has been used to illustrate the determination of gene regulations that were non-inferable from single-gene KO/KD experiments. A new performance score was introduced based on a redefinition of the confusion matrix, by taking into consideration non-inferable gene regulations. While the new assessment identified the same best performing teams as in the DREAM 4 inference challenge, the overall team rankings showed a prominent change from the original scoring. Furthermore, we showed that the majority of FP and FN errors among good performing inference methods could be explained to a great extent by the non-inferable part of the GRN. We also demonstrated that non-inferable regulations constituted the majority of common FNs among submissions in the 100-gene subchallenge. Therefore, it is crucial to consider network inferability in a fair and relevant assessment of network inference algorithms.

## Supporting Information

File S1 **MATLAB codes for the new assessment of network inference methods.**
(ZIP)

Figure S1 **Flowchart for calculation of the AUROC and AUPR in the new assessment.** Adapted from Figure A1 in [3].
(PDF)

Figure S2 **Comparison of the team scores between the new and the original assessment.** The two assessment procedures are applied to the 10-gene subchallenge (left) and the 100-gene subchallenge (right) of the DREAM 4 In Silico Network Challenge.
(PDF)

Table S1 **Results based on the new assessment of the 10-gene subchallenge of the DREAM 4 In Silico Network Challenge.**
(PDF)

Table S2 **Results based on the new assessment of the 100-gene subchallenge of the DREAM 4 In Silico Network Challenge.**
(PDF)

Table S3 **Fraction of FPs that are non-inferable from single-gene KO/KD experiments.** The fraction of FPs is calculated for each participant for networks 1 to 5 of the 100-gene subchallenge of the DREAM 4 In Silico Network Challenge.
(PDF)

Table S4 **Fraction of FNs that are non-inferable from single-gene KO/KD experiments.** The fraction of FNs is calculated for each participant for networks 1 to 5 of the 100-gene subchallenge of the DREAM 4 In Silico Network Challenge.
(PDF)
