# Supplementary figures and images for: Assessment of Network Inference Methods: How to Cope with an Underdetermined Problem
Source: PLoS One. 2014 Mar 6;9(3):e90481. doi: 10.1371/journal.pone.0090481 (PMC3946176; doi:10.1371/journal.pone.0090481)

Figure S1. Flowchart for calculation of AUROC and AUPR in the new assessment.

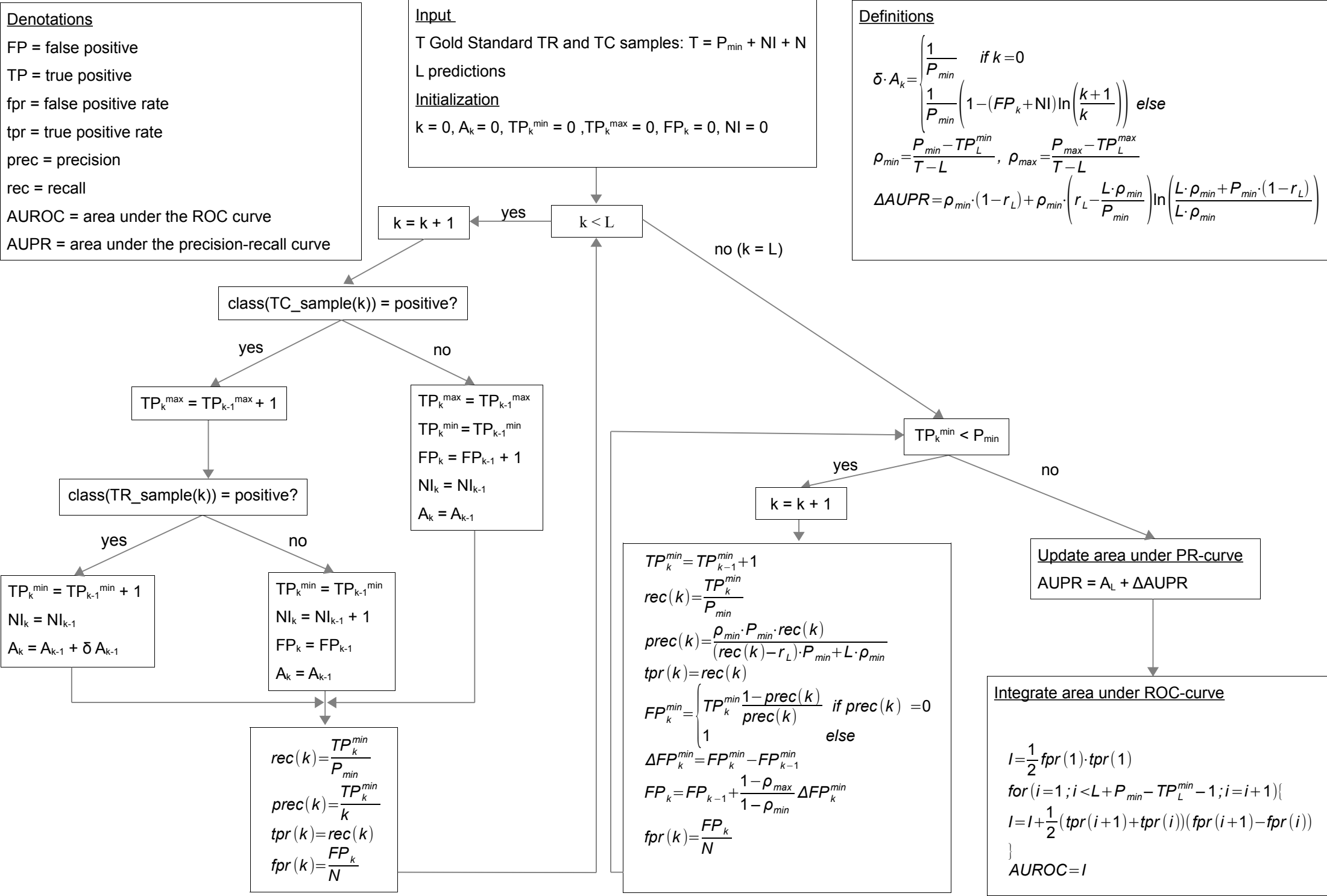

Supplement: Figure S1 — Flowchart for calculation of the AUROC and AUPR in the new assessment. Adapted from Figure A1 in [3]. (PDF) [file pone.0090481.s002.pdf]

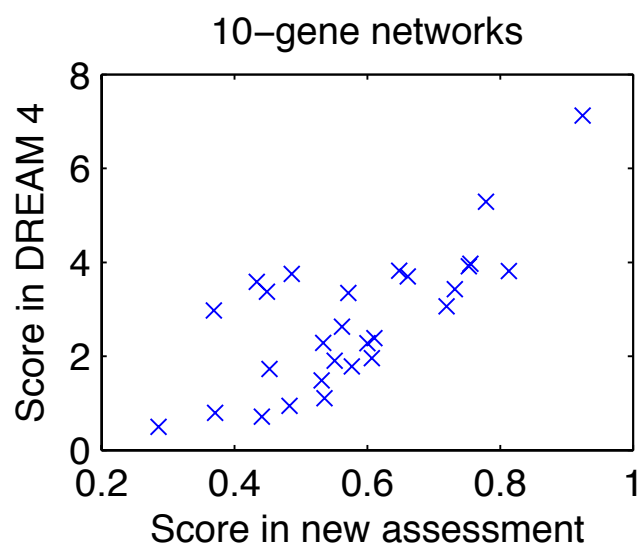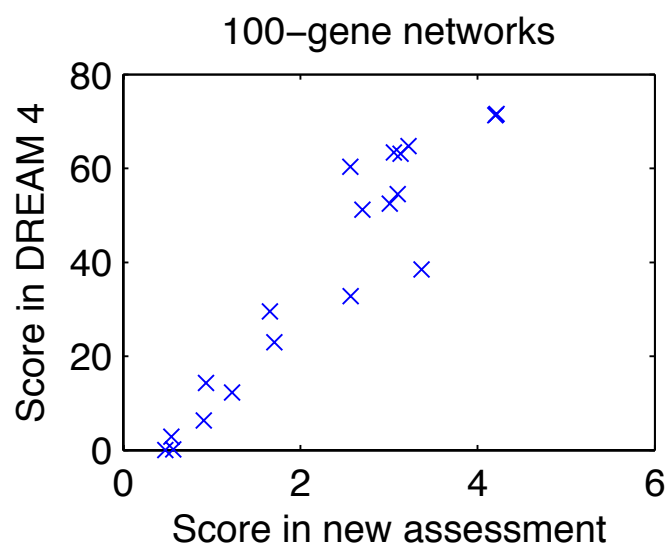

Figure S2. Comparison of team scores between the new and the original assessment.

Supplement: Figure S2 — Comparison of the team scores between the new and the original assessment. The two assessment procedures are applied to the 10-gene subchallenge (left) and the 100-gene subchallenge (right) of the DREAM 4 In Silico Network Challenge. (PDF) [file pone.0090481.s003.pdf]
