# Supplementary material for: Assessment of Network Inference Methods: How to Cope with an Underdetermined Problem
Source: PLoS One. 2014 Mar 6;9(3):e90481. doi: 10.1371/journal.pone.0090481 (PMC3946176; doi:10.1371/journal.pone.0090481)
Supplement: Table S1 — Results based on the new assessment of the 10-gene subchallenge of the DREAM 4 In Silico Network Challenge. (PDF) [file pone.0090481.s004.pdf]

Table S1. Results based on the new assessment of the 10-gene subchallenge of the DREAM 4 In Silico Network Challenge.

Rank		SCORE			AUROC Score					AUPR Score					AUROC					AUPR				
NEW	D4	OVERALL	AUROC	AUPR	Net 1	Net 2	Net 3	Net 4	Net 5	Net 1	Net 2	Net 3	Net 4	Net 5	Net 1	Net 2	Net 3	Net 4	Net 5	Net 1	Net 2	Net 3	Net 4	Net 5
1	1	0.924	0.973	0.876	1.111	0.779	1.000	0.965	1.008	1.869	0.072	0.039	1.250	1.148	0.986	0.779	1.000	0.920	0.895	0.919	0.048	0.039	0.716	0.539
2	6	0.813	0.963	0.663	1.118	0.756	1.000	1.049	0.893	1.597	0.040	0.027	0.680	0.971	0.991	0.756	1.000	1.000	0.793	0.785	0.026	0.027	0.389	0.456
3	2	0.779	0.977	0.581	1.127	0.814	1.000	1.049	0.893	2.034	0.064	0.032	0.497	0.277	1.000	0.814	1.000	1.000	0.793	1.000	0.043	0.032	0.285	0.130
4	3	0.755	0.854	0.656	1.088	0.581	1.000	0.895	0.708	1.438	0.038	0.189	0.297	1.317	0.965	0.581	1.000	0.853	0.628	0.707	0.025	0.189	0.170	0.619
5	4	0.753	0.828	0.679	1.127	0.419	0.871	1.049	0.672	1.723	0.023	0.018	1.343	0.286	1.000	0.419	0.871	1.000	0.596	0.847	0.015	0.018	0.769	0.134
6	10	0.732	0.859	0.605	1.124	0.291	0.871	1.049	0.960	1.380	0.021	0.014	1.250	0.357	0.997	0.291	0.871	1.000	0.853	0.678	0.014	0.014	0.716	0.168
7	13	0.719	0.866	0.573	1.127	0.349	1.000	1.049	0.802	1.460	0.026	0.027	1.093	0.259	1.000	0.349	1.000	1.000	0.712	0.718	0.018	0.027	0.626	0.122
8	8	0.661	0.740	0.582	1.127	0.640	0.129	1.049	0.755	1.960	0.030	0.006	0.619	0.297	1.000	0.640	0.129	1.000	0.670	0.964	0.020	0.006	0.355	0.139
9	5	0.648	0.849	0.447	1.118	0.419	1.000	1.035	0.676	1.279	0.023	0.088	0.373	0.471	0.991	0.419	1.000	0.987	0.600	0.629	0.015	0.088	0.214	0.221
10	16	0.611	0.888	0.333	1.078	0.895	0.903	1.007	0.557	1.112	0.076	0.021	0.349	0.107	0.957	0.895	0.903	0.960	0.495	0.547	0.051	0.021	0.200	0.050
11	19	0.607	0.845	0.369	1.088	0.884	0.742	0.755	0.755	0.817	0.277	0.019	0.112	0.620	0.965	0.884	0.742	0.720	0.670	0.402	0.184	0.019	0.064	0.292
12	18	0.600	0.369	0.831	0.082	0.453	0.484	0.063	0.765	2.034	0.026	0.008	1.746	0.342	0.072	0.453	0.484	0.060	0.679	1.000	0.017	0.008	1.000	0.160
13	21	0.577	0.716	0.437	0.869	0.703	0.452	0.846	0.711	1.039	0.044	0.007	0.768	0.328	0.771	0.703	0.452	0.807	0.632	0.511	0.029	0.007	0.440	0.154
14	12	0.571	0.761	0.381	1.088	0.640	0.161	0.993	0.925	0.947	0.061	0.007	0.207	0.684	0.965	0.640	0.161	0.947	0.821	0.466	0.041	0.007	0.119	0.321
15	15	0.562	0.575	0.550	0.943	0.535	0.355	0.629	0.411	1.229	0.346	0.058	0.067	1.049	0.836	0.535	0.355	0.600	0.365	0.604	0.230	0.058	0.038	0.493
16	20	0.551	0.579	0.523	0.863	0.750	0.500	0.021	0.759	0.425	0.085	0.008	1.746	0.353	0.765	0.750	0.500	0.020	0.674	0.209	0.056	0.008	1.000	0.166
17	24	0.535	0.790	0.281	0.820	0.628	0.871	0.699	0.933	0.950	0.045	0.028	0.075	0.305	0.728	0.628	0.871	0.667	0.828	0.467	0.030	0.028	0.043	0.143
18	17	0.534	0.678	0.389	0.706	0.698	0.355	1.028	0.605	0.570	0.045	0.008	0.878	0.444	0.626	0.698	0.355	0.980	0.537	0.280	0.030	0.008	0.503	0.209
19	23	0.531	0.672	0.391	0.830	0.395	0.452	0.829	0.854	0.642	0.024	0.008	0.662	0.618	0.736	0.395	0.452	0.790	0.758	0.316	0.016	0.008	0.379	0.290
20	7	0.486	0.384	0.589	0.229	0.680	0.194	0.126	0.694	1.960	0.039	0.137	0.346	0.460	0.203	0.680	0.194	0.120	0.616	0.964	0.026	0.137	0.198	0.216
21	25	0.483	0.791	0.174	0.905	0.791	0.871	0.587	0.802	0.508	0.101	0.052	0.052	0.158	0.803	0.791	0.871	0.560	0.712	0.250	0.067	0.052	0.030	0.074
22	22	0.453	0.679	0.227	0.624	1.000	0.484	0.650	0.636	0.122	0.292	0.012	0.130	0.579	0.554	1.000	0.484	0.620	0.565	0.060	0.194	0.012	0.074	0.272
23	11	0.449	0.359	0.538	0.016	0.477	0.500	0.021	0.783	1.869	0.025	0.008	0.496	0.293	0.014	0.477	0.500	0.020	0.695	0.919	0.017	0.008	0.284	0.138
24	27	0.442	0.756	0.127	0.444	0.837	0.903	0.790	0.806	0.089	0.108	0.034	0.208	0.195	0.394	0.837	0.903	0.753	0.716	0.044	0.072	0.034	0.119	0.092
25	9	0.434	0.432	0.435	0.503	0.453	0.452	0.448	0.304	1.362	0.281	0.058	0.279	0.198	0.446	0.453	0.452	0.427	0.270	0.670	0.187	0.058	0.160	0.093
26	26	0.371	0.593	0.149	0.529	0.523	0.194	0.794	0.923	0.106	0.033	0.017	0.111	0.479	0.470	0.523	0.194	0.757	0.819	0.052	0.022	0.017	0.064	0.225
27	14	0.369	0.277	0.461	0.049	0.477	0.000	0.105	0.755	1.358	0.026	0.019	0.292	0.611	0.043	0.477	0.000	0.100	0.670	0.667	0.017	0.019	0.167	0.287
28	28	0.286	0.497	0.074	0.570	0.477	0.355	0.542	0.543	0.123	0.065	0.027	0.068	0.089	0.506	0.477	0.355	0.517	0.482	0.060	0.044	0.027	0.039	0.042

D4: DREAM 4 assessment
