# Supplementary material for: Assessment of Network Inference Methods: How to Cope with an Underdetermined Problem
Source: PLoS One. 2014 Mar 6;9(3):e90481. doi: 10.1371/journal.pone.0090481 (PMC3946176; doi:10.1371/journal.pone.0090481)
Supplement: Table S2 — Results based on the new assessment of the 100-gene subchallenge of the DREAM 4 In Silico Network Challenge. (PDF) [file pone.0090481.s005.pdf]

Table S2. Results based on the new assessment of the 100-gene subchallenge of the DREAM 4 In Silico Network Challenge.

Rank		SCORE			AUROC Score					AUPR Score					AUROC					AUPR				
NEW	D4	OVERALL	AUROC	AUPR	Net 1	Net 2	Net 3	Net 4	Net 5	Net 1	Net 2	Net 3	Net 4	Net 5	Net 1	Net 2	Net 3	Net 4	Net 5	Net 1	Net 2	Net 3	Net 4	Net 5
1	1	4.216	1.351	7.080	1.530	1.388	1.376	1.180	1.283	17.819	6.640	4.370	1.454	5.116	0.906	0.852	0.895	0.757	0.786	0.411	0.163	0.146	0.046	0.125
2	2	4.201	1.368	7.033	1.529	1.413	1.359	1.208	1.331	17.140	7.260	4.741	1.344	4.682	0.906	0.868	0.884	0.775	0.815	0.395	0.179	0.158	0.042	0.114
3	10	3.366	1.183	5.550	1.428	1.214	1.182	1.061	1.028	12.387	6.493	3.256	4.028	1.588	0.846	0.745	0.769	0.681	0.630	0.286	0.160	0.108	0.127	0.039
4	3	3.221	1.285	5.158	1.490	1.447	1.283	1.089	1.115	12.129	7.917	3.722	0.830	1.191	0.883	0.889	0.835	0.698	0.683	0.280	0.195	0.124	0.026	0.029
5	5	3.130	1.374	4.886	1.546	1.353	1.413	1.246	1.310	12.820	5.611	3.863	0.989	1.146	0.915	0.831	0.919	0.799	0.802	0.296	0.138	0.129	0.031	0.028
6	7	3.099	1.242	4.957	1.383	1.251	1.172	1.183	1.221	10.005	6.739	2.778	1.222	4.041	0.819	0.768	0.763	0.759	0.748	0.231	0.166	0.093	0.038	0.099
7	4	3.057	1.343	4.770	1.505	1.368	1.361	1.192	1.290	9.352	6.619	6.023	0.687	1.171	0.891	0.840	0.885	0.764	0.790	0.216	0.163	0.201	0.022	0.029
8	8	3.009	1.377	4.641	1.554	1.445	1.348	1.204	1.334	10.049	4.157	2.882	1.201	4.914	0.920	0.888	0.877	0.772	0.817	0.232	0.102	0.096	0.038	0.120
9	9	2.701	1.277	4.125	1.440	1.289	1.331	1.143	1.181	8.612	6.279	4.197	0.570	0.966	0.853	0.792	0.866	0.733	0.723	0.199	0.155	0.140	0.018	0.024
10	11	2.567	1.293	3.842	1.353	1.322	1.392	1.113	1.284	3.702	4.470	5.441	1.182	4.417	0.801	0.812	0.905	0.714	0.786	0.085	0.110	0.181	0.037	0.108
11	6	2.565	1.248	3.883	1.452	1.320	1.286	1.061	1.120	7.299	5.836	2.384	0.693	3.203	0.860	0.811	0.837	0.680	0.686	0.168	0.144	0.079	0.022	0.078
12	13	1.706	1.138	2.274	1.265	1.110	1.239	1.045	1.032	4.405	2.806	2.676	0.581	0.904	0.749	0.682	0.806	0.670	0.632	0.102	0.069	0.089	0.018	0.022
13	12	1.654	1.167	2.142	1.375	1.201	1.176	1.036	1.047	4.776	1.454	0.927	0.312	3.241	0.815	0.738	0.765	0.664	0.641	0.110	0.036	0.031	0.010	0.079
14	15	1.229	1.000	1.458	1.204	1.031	0.928	0.885	0.950	4.676	1.230	0.503	0.283	0.597	0.713	0.633	0.604	0.568	0.582	0.108	0.030	0.017	0.009	0.015
15	14	0.936	1.083	0.789	1.244	1.113	1.187	0.883	0.990	1.720	1.144	0.507	0.164	0.410	0.737	0.684	0.772	0.566	0.606	0.040	0.028	0.017	0.005	0.010
16	16	0.908	1.006	0.811	0.984	1.014	0.987	1.005	1.040	1.064	0.925	0.278	1.144	0.641	0.583	0.623	0.642	0.645	0.637	0.025	0.023	0.009	0.036	0.016
17	18	0.564	0.833	0.296	0.922	0.890	0.646	1.006	0.699	0.482	0.268	0.088	0.424	0.219	0.546	0.547	0.421	0.645	0.428	0.011	0.007	0.003	0.013	0.005
18	17	0.543	0.818	0.268	0.952	0.773	0.715	0.799	0.849	0.539	0.223	0.139	0.164	0.275	0.564	0.475	0.465	0.513	0.520	0.012	0.005	0.005	0.005	0.007
19	19	0.476	0.750	0.202	0.805	0.745	0.685	0.762	0.754	0.383	0.209	0.088	0.119	0.214	0.476	0.457	0.446	0.489	0.462	0.009	0.005	0.003	0.004	0.005

D4: DREAM 4 assessment
