# Supplementary material for: Assessment of Network Inference Methods: How to Cope with an Underdetermined Problem
Source: PLoS One. 2014 Mar 6;9(3):e90481. doi: 10.1371/journal.pone.0090481 (PMC3946176; doi:10.1371/journal.pone.0090481)
Supplement: Table S3 — Fraction of FPs that are non-inferable from single-gene KO/KD experiments. The fraction of FPs is calculated for each participant for networks 1 to 5 of the 100-gene subchallenge of the DREAM 4 In Silico Network Challenge. (PDF) [file pone.0090481.s006.pdf]

Table S3. Fraction of FPs that are non-inferable from single-gene KO/KD experiments.

New Rank	Rank DREAM assessment	Net 1	Net 2	Net 3	Net 4	Net 5	Mean	Std
1	1	0.671	0.385	0.762	0.776	0.606	0.640	0.159
2	2	0.878	0.386	0.788	0.855	0.907	0.763	0.215
3	10	0.691	0.278	0.653	0.800	0.732	0.631	0.205
4	3	0.383	0.120	0.539	0.458	0.631	0.426	0.195
5	5	0.949	0.366	0.759	0.897	0.575	0.709	0.241
6	7	0.165	0.220	0.640	0.443	0.496	0.393	0.197
7	4	0.897	0.321	0.793	0.878	0.722	0.722	0.235
8	8	0.267	0.164	0.374	0.390	0.352	0.309	0.094
9	9	0.761	0.213	0.714	0.763	0.721	0.634	0.237
10	11	0.029	0.046	0.286	0.146	0.152	0.132	0.103
11	6	0.815	0.324	0.768	0.707	0.785	0.680	0.203
12	13	0.007	0.021	0.370	0.213	0.204	0.163	0.151
13	12	0.547	0.254	0.678	0.400	0.259	0.428	0.185
14	15	0.121	0.128	0.293	0.397	0.387	0.265	0.135
15	14	0.051	0.091	0.316	0.201	0.180	0.168	0.103
16	16	0.037	0.049	0.140	0.249	0.094	0.114	0.086
17	18	0.260	0.275	0.000	0.390	0.000	0.185	0.176
18	17	0.018	0.025	0.227	0.181	0.087	0.108	0.093
19	19	0.041	0.053	0.227	0.188	0.021	0.106	0.094
