# Supplementary material for: Assessment of Network Inference Methods: How to Cope with an Underdetermined Problem
Source: PLoS One. 2014 Mar 6;9(3):e90481. doi: 10.1371/journal.pone.0090481 (PMC3946176; doi:10.1371/journal.pone.0090481)
Supplement: Table S4 — Fraction of FNs that are non-inferable from single-gene KO/KD experiments. The fraction of FNs is calculated for each participant for networks 1 to 5 of the 100-gene subchallenge of the DREAM 4 In Silico Network Challenge. (PDF) [file pone.0090481.s007.pdf]

Table S4. Fraction of FNs that are non-inferable from single-gene KO/KD experiments.

New Rank	Rank DREAM assessment	Net 1	Net 2	Net 3	Net 4	Net 5	Mean	Std
1	1	0.549	0.830	0.842	0.808	0.712	0.748	0.122
2	2	0.524	0.841	0.856	0.769	0.707	0.739	0.134
3	10	0.505	0.815	0.864	0.785	0.698	0.733	0.141
4	3	0.519	0.808	0.861	0.780	0.688	0.731	0.134
5	5	0.532	0.821	0.848	0.786	0.688	0.735	0.129
6	7	0.524	0.780	0.808	0.800	0.641	0.711	0.124
7	4	0.517	0.814	0.856	0.772	0.665	0.725	0.136
8	8	0.533	0.805	0.870	0.772	0.710	0.738	0.128
9	9	0.511	0.813	0.857	0.771	0.682	0.727	0.137
10	11	0.493	0.804	0.902	0.810	0.735	0.749	0.155
11	6	0.489	0.824	0.857	0.767	0.696	0.727	0.146
12	13	0.522	0.805	0.884	0.794	0.648	0.731	0.144
13	12	0.430	0.785	0.825	0.794	0.684	0.703	0.162
14	15	0.511	0.790	0.832	0.810	0.685	0.726	0.133
15	14	0.481	0.782	0.819	0.810	0.674	0.713	0.142
16	16	0.472	0.781	0.820	0.827	0.707	0.722	0.147
17	18	0.468	0.758	0.821	0.834	0.689	0.714	0.149
18	17	0.461	0.758	0.818	0.814	0.683	0.707	0.148
19	19	0.477	0.760	0.820	0.822	0.688	0.713	0.143
